# Clinical variable-based cluster analysis identifies novel subgroups with a distinct genetic signature, lipidomic pattern and cardio-renal risks in Asian patients with recent-onset type 2 diabetes

**DOI:** 10.1007/s00125-022-05741-2

**Published:** 2022-06-28

**Authors:** Jiexun Wang, Jian-Jun Liu, Resham L. Gurung, Sylvia Liu, Janus Lee, Yiamunaa M, Keven Ang, Yi Ming Shao, Justin I-Shing Tang, Peter I. Benke, Federico Torta, Markus R. Wenk, Subramaniam Tavintharan, Wern Ee Tang, Chee Fang Sum, Su Chi Lim

**Affiliations:** 1 Clinical Research Unit, Khoo Teck Puat Hospital, Singapore, Republic of Singapore; 2 Department of Medicine, Khoo Teck Puat Hospital, Singapore, Republic of Singapore; 3 Lipidomics Incubator, Yong Loo Lin School of Medicine, National University of Singapore, Singapore, Republic of Singapore; 4 Diabetes Centre, Admiralty Medical Centre, Singapore, Republic of Singapore; 5 National Healthcare Group Polyclinic, Singapore, Republic of Singapore; 6 Saw Swee Hock School of Public Health, National University of Singapore, Singapore, Republic of Singapore; 7 Lee Kong Chian School of Medicine, Nanyang Technological University, Singapore, Republic of Singapore

**Keywords:** Beta cell dysfunction, Cardiovascular disease, Chronic kidney disease, Cluster analysis, Heart failure, Lipidomics, Mortality, Polygenic risk score, Type 2 diabetes mellitus

## Abstract

**Aims/hypothesis:**

We sought to subtype South East Asian patients with type 2 diabetes by de novo cluster analysis on clinical variables, and to determine whether the novel subgroups carry distinct genetic and lipidomic features as well as differential cardio-renal risks.

**Methods:**

Analysis by k-means algorithm was performed in 687 participants with recent-onset diabetes in Singapore. Genetic risk for beta cell dysfunction was assessed by polygenic risk score. We used a discovery–validation approach for the lipidomics study. Risks for cardio-renal complications were studied by survival analysis.

**Results:**

Cluster analysis identified three novel diabetic subgroups, i.e. mild obesity-related diabetes (MOD, 45%), mild age-related diabetes with insulin insufficiency (MARD-II, 36%) and severe insulin-resistant diabetes with relative insulin insufficiency (SIRD-RII, 19%). Compared with the MOD subgroup, MARD-II had a higher polygenic risk score for beta cell dysfunction. The SIRD-RII subgroup had higher levels of sphingolipids (ceramides and sphingomyelins) and glycerophospholipids (phosphatidylethanolamine and phosphatidylcholine), whereas the MARD-II subgroup had lower levels of sphingolipids and glycerophospholipids but higher levels of lysophosphatidylcholines. Over a median of 7.3 years follow-up, the SIRD-RII subgroup had the highest risks for incident heart failure and progressive kidney disease, while the MARD-II subgroup had moderately elevated risk for kidney disease progression.

**Conclusions/interpretation:**

Cluster analysis on clinical variables identified novel subgroups with distinct genetic, lipidomic signatures and varying cardio-renal risks in South East Asian participants with type 2 diabetes. Our study suggests that this easily actionable approach may be adapted in other ethnic populations to stratify the heterogeneous type 2 diabetes population for precision medicine.

**Graphical abstract:**

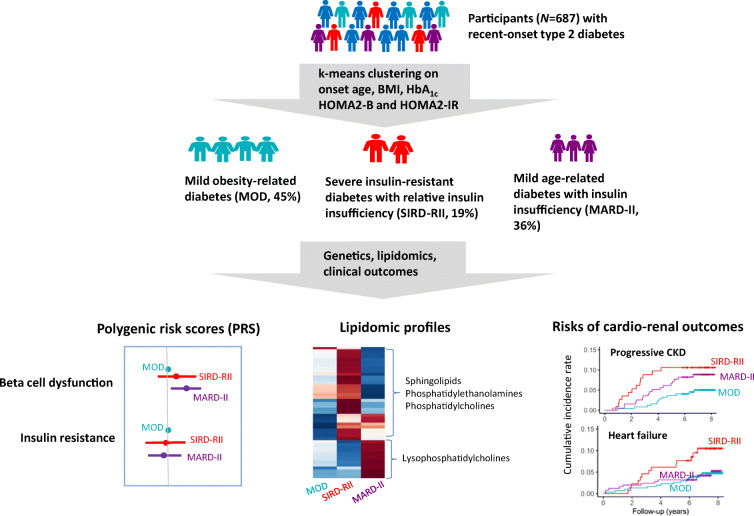

**Supplementary Information:**

The online version contains peer-reviewed but unedited supplementary material available at 10.1007/s00125-022-05741-2.

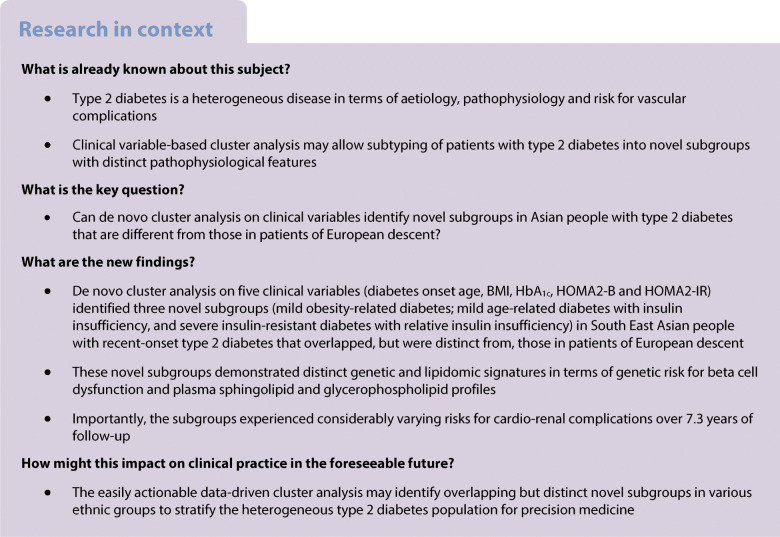



## Introduction

The pathogenesis of type 2 diabetes involves a complex interplay between genetic susceptibility and environmental factors [1–3]. Comorbidities such as obesity and dyslipidaemia often co-exist with dysregulation of glucose metabolism. Hence, type 2 diabetes is highly heterogeneous in terms of aetiology, clinical presentation, and risks for vascular and non-vascular complications [4–7]. However, patients with type 2 diabetes may be subtyped into relatively homogenous subgroups for precision medicine [1].

In a landmark study using data-driven cluster analysis, Ahlqvist et al subtyped recent-onset diabetes into five subgroups based on common clinical variables under the assumption that diabetes is clinically manifested when insulin secretion does not match decreased sensitivity [4, 8]. This novel but easily actionable subtyping approach has attracted tremendous interest, and the clustering algorithm has been replicated in several diabetic populations in recent years [9–13]. While cluster analysis on only a few clinical variables has the advantage of simplicity compared with other approaches using omics data [3], it may be argued that clusters identified from a data correlation matrix are simply the result of inter-dependency in the clinical variables [14]. One approach to address this concern is to examine whether the clusters derived from the common clinical variable have shared pathophysiological features within the subgroup but distinct from the other subgroups. Indeed, a recent study showed that inflammation biomarkers differed greatly across the novel subgroups [15]. Other studies in a European population also identified diabetic subgroups that differed in genetic risk, lipidomic and proteomic signatures [16, 17].

Compared with patients of European descent, Asians with type 2 diabetes have more severe adiposity at the same level of BMI, develop diabetes at a younger age, and demonstrate impaired insulin secretion to compensate for insulin resistance [18, 19]. South East Asia has a large population with type 2 diabetes due to the dramatic socioeconomic transition over recent decades [20]. However, to our knowledge, data on clinical variable-based cluster analysis from this region are still scarce. Most early replication studies used cluster coordinates derived from the ANDIS cohort (All New Diabetics in Scania) [8], rather than de novo cluster analysis, to subtype type 2 diabetes [12, 13, 21–23]. We hypothesise that analysis on the same set of clinical variables used in the ANDIS cohort may identify novel subgroups in Asian patients that differ from those in patients of European descent.

In the current study, we performed de novo cluster analysis in patients with recent-onset type 2 diabetes in Singapore, a city state in South East Asia with a mix of three major ethnic populations. We wished to determine whether the newly identified subgroups differ in aetiology and pathophysiology from the perspective of genetics and lipidomics. Importantly, we sought to determine whether these novel subgroups predict risks for cardio-renal complications over long-term follow-up.

## Methods

### Research design

We focused on individuals with a diabetes duration of less than 5 years in the current study because subgroup assignment derived from cluster analysis has been shown to be relatively stable within 5 years after diabetes onset [12]. Details of the cohort used (SMART2D, Singapore Study of Macro-Angiopathy and Micro-Vascular Reactivity in Type 2 Diabetes) have been described elsewhere [24]. Briefly, 2057 participants with type 2 diabetes were recruited from outpatient clinics in a secondary hospital and an adjacent primary care medical facility between 2011 and 2014. Type 2 diabetes was diagnosed by the attending physicians after excluding type 1 diabetes and diabetes attributable to specific causes. Type 1 diabetes was diagnosed as sustained requirement for insulin treatment within 1 year after diabetes diagnosis without measurement of GAD antibody. Patients with cancer and autoimmune disease on active treatments, and those with HbA_1c_ >12% (108 mmol/mol) at screening were also excluded from the cohort. Participants were recalled for a research visit every 3 years, and also followed up by reviewing electronic health records [25]. All 687 participants with diabetes duration ≤5 years and eGFR ≥15 ml min^–1^ 1.73 m^–2^ were included in the current analysis.

This study was approved by the Singapore National Healthcare Group Domain Specific Review Board and all participants provided written informed consent.

### Clinical and biochemical variables

Diabetes duration was self-reported. Blood pressure was measured three times using a semi-automated blood pressure monitor, and the mean value was used. Fasting plasma glucose, HDL- and LDL-cholesterol and triacylglycerols were quantified by enzymatic methods (Roche Cobas Integra 700; Roche Diagnostics, Basel, Switzerland). HbA_1c_ was measured using a point-of-care analyser (DCA Vantage Analyzer; Siemens, Munich, Germany). Serum creatinine was measured using an enzymatic method, and GFR was estimated using the CKD-EPI equation [26]. Urinary albumin was quantified using an immunoturbidimetric assay (Roche Cobas c, Roche Diagnostics, Mannheim, Germany). Plasma C-reactive protein was quantified using an immunoassay kit (R&D Systems, Minneapolis, MN, USA). Fasting plasma C-peptide was measured using an ELISA kit (Mercodia, Uppsala, Sweden). Both intra- and inter-assay CVs were <5%. HOMA2-B (%) and HOMA2-IR were calculated based on fasting glucose and C-peptide (https://www.dtu.ox.ac.uk/homacalculator/, version 2.2.3).

### Cluster analysis

We applied the k-means algorithm as proposed by Ahlqvist et al to divide participants into subgroups [8]. Five clinical classifiers (diabetes onset age, BMI, HbA_1c_, log-transformed HOMA2-B and HOMA2-IR) were standardised to a mean value of 0 and SD of 1. The optimal number of clusters was determined by majority voting according to 26 indices provided by the R package ‘NbClust’. Cluster stability was assessed by the Jaccard index based on bootstrapping [27].

### Beta cell dysfunction, insulin resistance and type 1 diabetes polygenic risk scores

Genome-wide association study (GWAS) and principal component analysis on GWAS arrays in participants of the cohort have been described before [28]. We created polygenic risk scores (PRSs) for beta cell dysfunction and insulin resistance based on 35 SNPs associated with insulin secretion and 20 SNPs associated with insulin sensitivity, respectively, in Asian populations. We weighted the SNPs by their effect on the risk of type 2 diabetes to determine whether the novel subgroups differ in genetic risk for type 2 diabetes development (see electronic supplementary material [ESM] Table 1) [29]. A high score indicates more severe beta cell dysfunction and insulin resistance. A type 1 diabetes PRS was constructed by a similar approach using nine SNPs (ESM Table 2) [30, 31]. Details on PRS derivation are given in ESM Methods. We fitted linear regression models to compare the differences in PRS across the three subgroups, in which the score was entered as a dependent variable and subgroup membership, sex and scores for the top three principal components in lieu of self-reported ethnicity were entered as covariates.

### Lipidomics assay and data analysis

We adopted a discovery–validation approach for the lipidomics study to reduce the likelihood of false positives due to multiple comparisons. The validation study was nested in an independent cohort that has been described previously [32]. In brief, 226 participants with diabetes duration ≤5 years and eGFR ≥15 ml min^–1^ 1.73 m^–2^ were randomly selected. As measurements of HOMA2-IR and HOMA2-B were not available for the validation cohort, we used the plasma triacylglycerol/HDL-cholesterol ratio as a proxy for insulin resistance [4, 33]. Using the ‘reference’ approach [4], the coordinate of the cluster centre in the discovery cohort was calculated as the mean value of BMI, diabetes onset age, HbA_1c_ and the plasma triacylglycerol/HDL-cholesterol ratio, and participants in the validation cohort were assigned cluster membership based on minimal Euclidean distance.

Technical details for the lipidomics assay by LC-MS are described in ESM Methods. A total of 315 lipid species were included in the discovery study after excluding those with a signal-to-noise ratio <3 and correcting for batch effect. We applied the Kruskal–Wallis test to compare the levels of lipid species across the three subgroups. Those with *p* values below the Bonferroni correction threshold (*p* <1.59 × 10^-4^, 0.05/315) were subjected to the Kruskal–Wallis test in the validation cohort, and a nominal *p* value <0.05 was considered statistically significant. We plotted a heatmap to visualise lipid species that differed across diabetes subgroups in both discovery and validation cohorts. Furthermore, we fitted linear regression models to compare differences in lipid species between two subgroups, in which log-transformed lipid concentration was entered as a dependent variable and subgroup membership as an independent variable.

#### Identification of adverse clinical outcomes and statistical analysis

All-cause mortality was identified from electronic medical records and cross-validated against the national death registry [34]. Cardiovascular death was identified from death certificates. Non-fatal acute myocardial infarction and stroke were identified from hospitalisation discharge summaries and surgical operation procedures. Major adverse cardiovascular events (MACE) were a composite of non-fatal acute myocardial infarction, stroke and death attributable to cardiovascular disease, whichever occurred first. Ascertainment of incident heart failure has been described previously [35]. Progressive chronic kidney disease (CKD) was defined as a decrease in eGFR of 40% or more from the baseline level, with repeated measurements at least 3 months apart as confirmation [36]. The follow-up was censored at 30 November 2019.

Incidence rates for progressive CKD, incident heart failure, MACE and all-cause mortality are presented as event number per 1000 person-years. The cumulative risk for cardio-renal events was plotted by the Kaplan–Meier approach and compared by logrank test. We fitted Cox proportional hazard regression models to study the associations of subgroup with cardio-renal risks in the follow-up period. Index age, sex and ethnicity were included as covariates in the models. We also adjusted baseline eGFR for the study on progressive CKD. The proportional hazards assumption was tested based on Schoenfeld residuals. No violation of proportional hazard assumption was identified.

## Results

### Data-driven cluster analysis identified three novel subgroups in participants with recent-onset type 2 diabetes

A total of 687 individuals with recent-onset diabetes were subjected to cluster analysis [8]. Majority voting according to 26 indices suggested that the study population may be optimally partitioned into three subgroups (ESM Fig. 1). The mean values of Jaccard indices were above 0.86 for all three clusters based on 5000 bootstraps, implying that the clusters were stable. Participant baseline characteristics in the three subgroups are presented in Table 1 and ESM Fig. 2.

**Table 1:** Baseline characteristics of three subgroups derived from k-means cluster analysis in individuals with recent-onset type 2 diabetes (*n*=687)

Variable	MOD (45%)	SIRD-RII (19%)	MARD-II (36%)	*p* value
Number of participants	307	130	250	–
Index age (years)	55 ± 9.6	43 ± 11.5	59 ± 9.9	<0.001
Age at diabetes onset (years)	52 ± 9.4	40 ± 10.9	56 ± 9.9	<0.001
Male	144 (46.9)	74 (56.9)	131 (52.4)	0.13
Ethnicity				<0.001
Chinese	123 (40.1)	62 (47.7)	157 (62.8)	
Malay	104 (33.9)	37 (28.5)	49 (19.6)	
Asian Indian	80 (26.1)	31 (23.8)	44 (17.6)	
Diabetes duration (years)	3.0 (1.0–4.0)	3.0 (2.0–5.0)	3.0 (1.0–5.0)	0.19
BMI (kg/m^2^)	30.1 ± 5.0	31.6 ± 5.9	24.9 ± 3.4	<0.001
HbA_1c_ (%)	6.9 ± 0.7	9.2 ± 1.2	7.1 ± 0.9	<0.001
HbA_1c_ (mmol/mol)	52 ± 5.3	77 ± 10	54 ± 6.8	–
Fasting plasma glucose (mmol/l)	6.5 ± 1.4	10.7 ± 2.6	7.4 ± 1.7	<0.001
Fasting C-peptide (pmol/l)	931 (771–1185)	920 (709–1305)	523 (386–644)	<0.001
HOMA2-B (%)	96.9 (77.5–131.7)	43.7 (32.4–61.9)	53.3 (40.0–66.0)	<0.001
HOMA2-IR	2.2 (1.8–2.9)	2.7 (1.9–3.8)	1.3 (0.9–1.6)	<0.001
Triacylglycerol/HDL ratio	1.1 (0.8–1.8)	1.6 (1.1–2.3)	0.9 (0.6–1.4)	<0.001
Blood pressure (mmHg)				
Systolic	137 ± 16.8	136 ± 14.9	139.3 ± 18.0	0.07
Diastolic	80.2 ± 9.3	82.0 ± 9.2	78.3 ± 9.5	0.001
Kidney function				
eGFR (ml min^–1^ 1.73 m^–2^)	92 ± 19.2	109 ± 19.7	92 ± 21.0	<0.001
Urine ACR (mg/mmol)	1.5 (0.5–4.1)	2.7 (0.7–8.4)	1.1 (0.1–4.6)	<0.001
Lipid profile, mmol/l				
HDL-cholesterol	1.3 ± 0.4	1.2 ± 0.3	1.4 ± 0.4	<0.001
LDL-cholesterol	2.8 ± 0.8	3.3 ± 0.9	2.8 ± 0.8	<0.001
Triacylglycerol	1.4 (1.1–1.9)	1.8 (1.3–2.4)	1.2 (0.9–1.6)	<0.001
C-reactive protein (μg/ml)	2.7 (1.1–5.8)	3.8 (2.0–7.9)	1.5 (0.5–3.5)	<0.001
Medication usage				
Metformin	243 (79.2)	118 (90.8)	204 (81.6)	0.02
Sulfonylurea	127 (41.4)	63 (48.5)	100 (40.0)	0.27
DPP4 inhibitor	7 (2.3)	10 (7.7)	6 (2.4)	0.01
Insulin	14 (4.6)	25 (19.2)	25 (10.0)	<0.001
Statins	232 (75.6)	92 (70.8)	183 (73.2)	0.49
RAS blocker	141 (45.9)	65 (50.0)	116 (46.4)	0.79

Data are presented as means ± SD, median (IQR) or *n* (%)

ACR, albumin/creatinine ratio; DPP4, dipeptidyl peptidase 4; RAS, renin–angiotensin system

Cluster 1 (45% of participants) was labelled as mild obesity-related diabetes (MOD). Participants in this subgroup had a high BMI (30.1 ± 5.0 kg/m^2^), an elevated HOMA2-IR (median 2.2, IQR 1.8–2.9) and preserved insulin secretion as evidenced by high levels of fasting C-peptide and HOMA2-B (median 97%, IQR 78–132%).

Cluster 2 (19.0% of participants) was labelled as severe insulin-resistant diabetes with relative insulin insufficiency (SIRD-RII). These participants had the highest level of HOMA2-IR (median 2.7, IQR 1.9–3.8), the highest BMI (31.6 ± 5.9 kg/m^2^) and the worst glycaemic control among the three subgroups. They also had the highest level of triacylglycerols, the lowest level of HDL-cholesterol, the highest level of C-reactive protein and the youngest age at diabetes diagnosis. Their HOMA2-B index was low (median 44%, IQR 32–62%) but their fasting C-peptide remained at a high level compared with the other two subgroups.

Cluster 3 (36% of participants) was labelled as mild age-related diabetes with insulin insufficiency (MARD-II). These participants were slightly older (56 ± 9.9 years) and had a low HOMA2-B index (median 53%, IQR 40–66%) at diabetes diagnosis. Their fasting C-peptide was 45% lower than the other two subgroups. They had no overt obesity (BMI 24.9 ± 3.4 kg/m^2^) and only moderately elevated HOMA2-IR (median 1.3, IQR 0.9–1.6).

The MOD subgroup was taken as the reference in the subsequent analyses because it was the largest subgroup in the study population.

### High PRS for beta cell dysfunction in the MARD-II subgroup

Compared with the MOD subgroup, the participants in the MARD-II subgroup had a significantly higher PRS for beta cell dysfunction after adjustment for sex and GWAS principal components 1–3 (Table 2). There was no significant difference in the PRS for beta cell dysfunction between the SIRD-RII and MOD subgroups, and no significant difference in the PRS for insulin resistance among the three subgroups.

**Table 2:** Association of polygenic risk scores with subgroup membership

PRS	Beta cell dysfunction coefficient (95% CI)	*p* value	Insulin resistance coefficient (95% CI)	*p* value
MOD	Reference		Reference	
SIRD-RII	1.00 (−0.31, 2.31)	0.14	−0.15 (−0.85, 0.56)	0.68
MARD-II	1.39 (0.32, 2.47)	0.01	−0.11 (−0.69, 0.46)	0.70
Male sex	Reference		Reference	
Female sex	1.30 (0.34, 2.25)	0.01	0.28 (−0.23, 0.79)	0.28

For the linear regression models, the PRS was the dependent variable and cluster membership (MOD subgroup as reference), sex and GWAS principal components 1–3 were used as covariates

### Distinct lipidomic patterns across the three subgroups

**Fig. 1:**
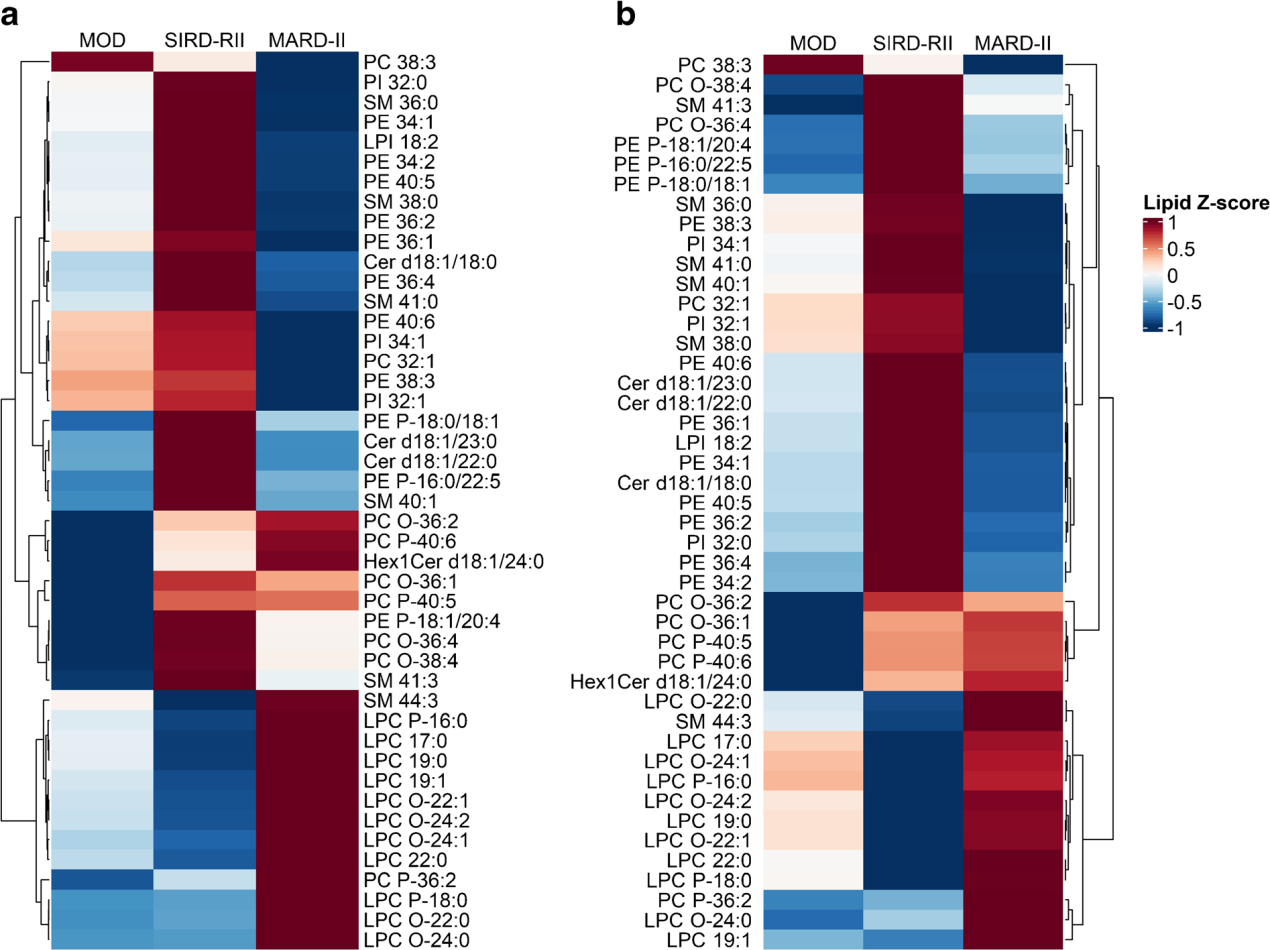
Plasma lipid species that differed significantly across the three subgroups in the discovery cohort (**a**) and the validation cohort (**b**). PC, phosphatidylcholine; PI, phosphatidylinositol; SM, sphingomyelin; PE, phosphatidylethanolamine; Cer, ceramide; LPC, lyso-phosphatidylcholine; LPI, lyso-phosphatidylinositol; Hex1Cer, monohexosylceramide

The clinical profiles of the discovery and validation cohorts were comparable (ESM Tables 3 and 4). Of the 315 lipid species included in the discovery study, 75 differed across the three subgroups (*p* value <1.59 × 10^-4^), and 45 of them also differed significantly across the three subgroups in the validation cohort (nominal *p* <0.05, ESM Fig. 3). The SIRD-RII subgroup had high levels of glycerophospholipids, mainly phosphatidylethanolamine, phosphatidylcholine and phosphatidylinositol subspecies, but lower levels of lysophosphatidylcholine (LPC), including subspecies with alkyl ether and plasmalogen bonds. They also had remarkably high level of sphingolipids (sphingomyelins and ceramides). In contrast, the MARD-II subgroup had low levels of glycerophospholipids and sphingolipids but higher levels of LPC (Fig. 1). The subsequent between-group comparisons identified 17 lipid species, mainly phosphatidylethanolamine, phosphatidylcholine, ceramides and sphingomyelins, that differed between the SIRD-RII and MOD subgroups. The phosphatidylethanolamine, phosphatidylinositol, phosphatidylcholine and sphingomyelin levels were lower in the MARD-II subgroup compared with the MOD subgroup, and levels of LPC subspecies were higher (ESM Tables 5 and 6).

### Risks for cardio-renal complications in the three subgroups during follow-up

The median follow-up duration was 7.3 years (IQR 6.7–7.7). The crude incident rates for progressive CKD, incident heart failure, MACE and all-cause mortality are shown in ESM Table 7. The incident rate for heart failure in the SIRD-RII subgroup (14.5 per 1000 person-years; 95% CI 7.7, 24.8) was twofold higher than that in the other subgroups: 6.4 for the MOD subgroup (95% CI 3.5, 10.7) and 6.3 for the MARD-II subgroup (95% CI 3.1, 11.2). Additionally, the SIRD-RII subgroup had the highest risk for progressive CKD, followed by the MARD-II and MOD subgroups: 15.9 (95% CI 8.2, 27.8), 12.7 (95% CI 7.4, 20.3) and 6.8 (95% CI 3.5, 11.9) per 1000 person-years, respectively.

**Fig. 2:**
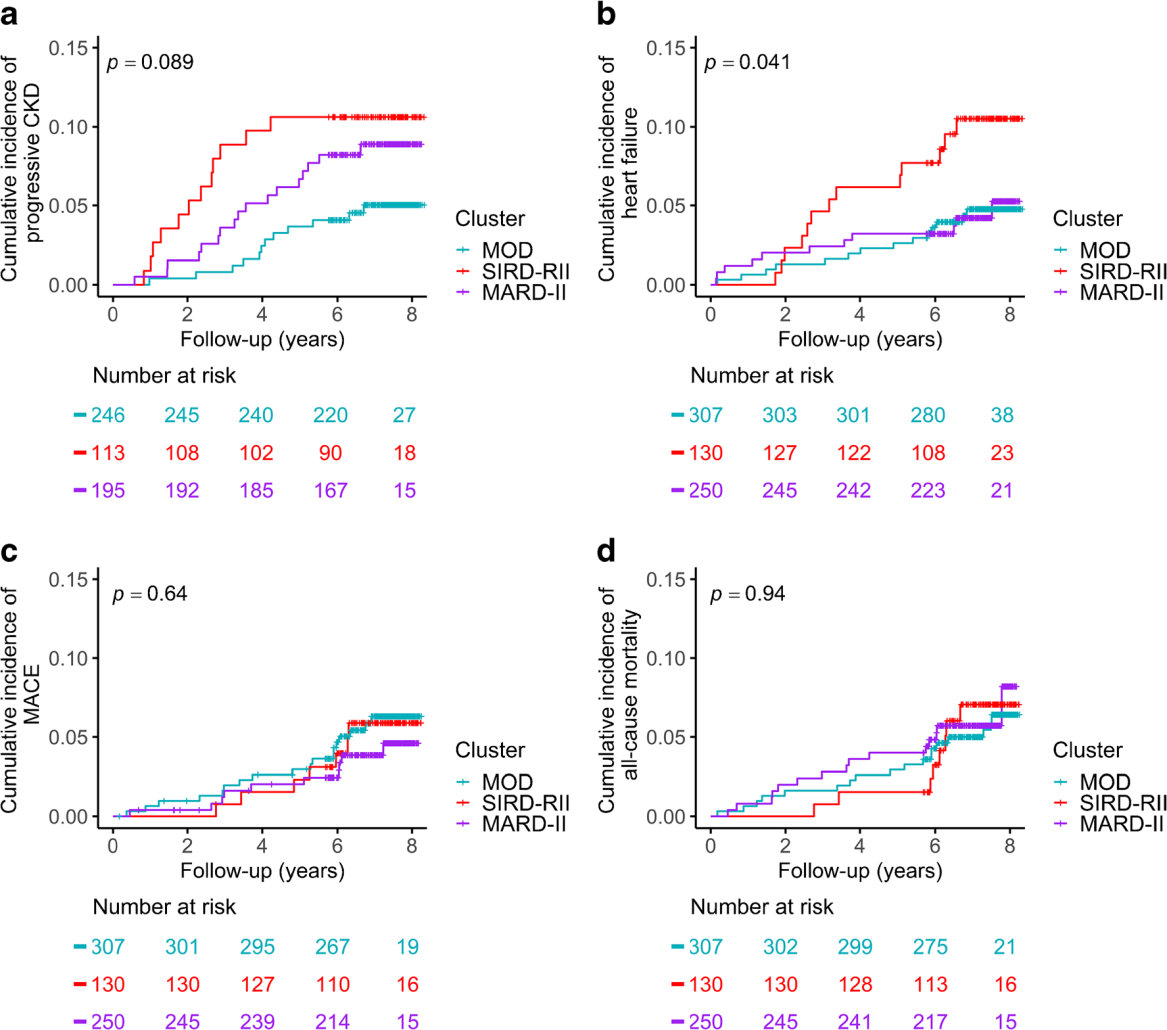
Cumulative incidence of adverse clinical outcomes by diabetes subgroups: (**a**) progressive CKD, (**b**) incident heart failure, (**c**) MACE, and (**d**) all-cause mortality. Only participants with baseline eGFR above 60 ml min 1.73 m^–2^ were included in the analysis on progressive CKD

Cumulative incidences were plotted for visualisation by the Kaplan–Meier approach (Fig. 2). Cox proportional hazard regression models suggested that the SIRD-RII subgroup had a 2.30-fold unadjusted risk (95% CI 1.08, 4.89) for heart failure compared with the MOD subgroup. Adjustment for index age, sex and ethnicity strengthened the association (adjusted HR 5.23; 95% CI 2.35, 11.60). The SIRD-RII subgroup had a 2.33-fold unadjusted risk (95% 1.05, 5.18) and a 3.67-fold adjusted risk (95% CI 1.53, 8.80) for progressive CKD, with adjusted and unadjusted hazard ratios of 1.84 (95% 0.88, 3.85) and 2.64 (95% CI 1.20, 5.82) in the MARD-II subgroup compared with the MOD subgroup. Additionally, the SIRD-RII subgroup had the same unadjusted risks for MACE and all-cause mortality as the other subgroups, despite being more than 10 years younger and having similar diabetes duration. Further analysis suggested that they had a 2.99-fold age-adjusted risk (95% CI 1.22, 7.30) for all-cause mortality compared with the MOD subgroup (Table 3).

**Table 3:** Association of novel subgroup with adverse cardio-renal risk during the follow-up period by Cox proportional hazard regression

Outcome	Unadjusted HR (95% CI)	*p* value	Adjusted HR^a^ (95% CI)	*p* value
Progressive CKD				
MOD	Reference		Reference	
SIRD-RII	2.33 (1.05, 5.18)	0.04	3.67 (1.53, 8.8)	0.004
MARD-II	1.84 (0.88, 3.85)	0.11	2.64 (1.20, 5.82)	0.02
Heart failure				
MOD	Reference		Reference	
SIRD-RII	2.30 (1.08, 4.89)	0.03	5.23 (2.35, 11.6)	<0.001
MARD-II	0.99 (0.45, 2.17)	0.97	0.87 (0.39, 1.98)	0.75
MACE				
MOD	Reference		Reference	
SIRD-RII	0.93 (0.39, 2.22)	0.87	1.92 (0.75, 4.89)	0.17
MARD-II	0.69 (0.32, 1.50)	0.35	0.61 (0.27, 1.36)	0.23
All-cause mortality				
MOD	Reference		Reference	
SIRD-RII	1.12 (0.48, 2.59)	0.80	2.99 (1.22, 7.30)	0.02
MARD-II	1.11 (0.56, 2.23)	0.76	0.78 (0.38, 1.62)	0.51

^a^Age, sex and ethnicity were adjusted for outcomes of all-cause mortality, incident heart failure and MACE in Cox proportional hazard regression models. Baseline eGFR was also adjusted for outcome of progressive CKD

### Additional analyses

To assess whether the participant’s sex affects cluster analysis, we regressed sex on clinical variables and used regression residuals as the new classifiers [37]. This new analysis also partitioned participants into three clusters, and the cluster membership showed high agreement with that in the primary analysis (approximately 90% concordance, ESM Table 8). We also clustered participants into four subgroups according to centroids derived from the ANDIS cohort [8]. As shown in ESM Figs 4 and 5, 67% of the participants in the severe insulin-deficient diabetes (SIDD) subgroup were from the SIRD-RII subgroup although they did not have a significantly lower level of fasting C-peptide. In the follow-up period, the SIDD subgroup had the highest risk for progressive CKD (ESM Table 9). This finding was different from that of previous studies, which showed that the SIRD group had the highest risk for progressive CKD [8, 10, 12, 23]. Participants with a type 1 diabetes PRS in the top five percentiles had a slightly lower HOMA2-B (57% vs 69%, *p*=0.02) compared with those in the remaining 95 percentiles. However, fasting C-peptide did not differ between the two groups (*p*=0.19). As shown in ESM Table 10, 9% and 7% participants in the SIRD-RII and MARD-II subgroups, respectively, were classified as having a high type 1 diabetes PRS.

## Discussion

By applying the k-means algorithm on the same clinical variables as proposed by the previous study in a European population [8], we identified three novel subgroups in participants with recent-onset type 2 diabetes in our South East Asian cohort. The largest subgroup (MOD, 45% of participants) is characterised by mild obesity, insulin resistance and preserved insulin secretion. The second largest subgroup (MARD-II, 36% of participants) is characterised by a slightly older age of diabetes onset and low beta cell secretion with no overt insulin resistance. The third subgroup (SIRD-RII, 19% of participants) is characterised by severe insulin resistance, poor glycaemic control and relative insulin insufficiency as indicated by preserved insulin secretion but a low HOMA2-B. Our genetics and lipidomics studies suggest a significant difference in genetic risks for diabetes aetiology and distinct pathophysiological features in the three subgroups. Importantly, we demonstrate that the clinical variable-based cluster analysis may potentially stratify patients by risk for cardio-renal complications after diabetes onset.

As hypothesised, we identified diabetes subgroups with overlapping but distinct characteristics in our South East Asian population compared with patients of European descent. In the landmark study by Ahlqvist et al [8], and also in subsequent replication studies in the European and US populations [10, 12, 21], the largest subgroup is MARD (approximately 40%), followed by the MOD subgroup (approximately 20%) and the SIRD subgroup (approximately 20%). In the present study, the largest subgroup is MOD (45%), followed by the MARD-II subgroup (36%). The dominance of obesity-related diabetes in this Asian study population may be attributable to the socioeconomic transformation and concurrent rapid increase in the prevalence of obesity over recent decades in this population. Our study indicates that de novo cluster analysis is warranted to subtype heterogeneous type 2 diabetes patients in various ethnic populations. As shown in ESM Fig. 5, patients in our SIDD subgroup derived from the ANDIS cohort centroids do indeed have a low HOMA2-B. However, their C-peptide level is close to the mean value of the full cohort, suggesting that these participants do not have insulin deficiency.

Elucidating the aetiology of diabetes may shed light on strategies for diabetes prevention and treatment. Our data suggest that obesity and the related insulin resistance are the main driving factor for diabetes pathogenesis in this Asian study population. This highlights the importance for prevention and treatment of obesity to slow down the rising prevalence of diabetes in this region [20]. However, the MARD-II subgroup has neither overt obesity nor insulin resistance. Instead, they are characterised by a 40% lower fasting C-peptide and a low HOMA2-B. These features are different in patients of European descent, in whom the HOMA2-B index in this subgroup remained high [8]. Patients in this subgroup have a higher PRS for beta cell dysfunction, which suggests the presence of impaired beta cell function determined by genetic background. This is in agreement with a large study showing that patients with type 2 diabetes who have a high number of beta cell dysfunction-related genetic loci have a reduced fasting C-peptide level [3].

Participants in the SIRD-RII subgroup had the highest HOMA2-IR and the worst glycaemic control. The high levels of BMI, triacylglycerol and C-reactive protein, low level of HDL-cholesterol, and the markedly increased levels of sphingo- and glycerophospholipids also support the presence of severe insulin resistance. The low HOMA2-B in this subgroup should be interpreted in the presence of high HOMA2-IR and uncontrolled hyperglycaemia. We reasoned that participants in this subgroup do not have absolute insulin deficiency because their fasting C-peptide was at a level comparable to that in the MOD subgroup and 40% higher than that in the MARD-II subgroup. Instead, the low level of HOMA2-B suggests that the beta cell secretion capacity is unable to adequately compensate for severe insulin resistance [2]. Hence, we designated them as having ‘relative insulin insufficiency’. This is in agreement with our genetic study, which did not find a higher PRS for beta cell dysfunction in this subgroup. We postulate that the relatively insufficient insulin secretion in this subgroup may be partly attributable to glucotoxicity and lipotoxicity, given the uncontrolled hyperglycaemia, overt dyslipidaemia and abnormal lipidomics profile. Intriguingly, we did not observe a significant difference in the PRS for insulin resistance across the three subgroups. This may suggest that genetic risk is not the main determinant for insulin resistance in the SIRD-RII subgroup. However, it may be more reasonable to attribute the null analytical outcome to the relatively small sample size in the current study.

Plasma lipidomic signatures have been associated not only with the risk for type 2 diabetes pathogenesis but also the risk for diabetic complications [38]. Compared with the MOD subgroup, the SIRD-RII subgroup is characterised by activation of the ceramide/sphingomyelin pathway and remodelling of glycerophospholipid metabolism, but the levels of these two classes of lipids were lower in the MARD-II subgroup. Both sphingolipid and glycerophospholipid metabolism have been associated with insulin resistance [39, 40], supporting the consistency between the lipidomic signature and clinical phenotype in the current study. On the other hand, LPC level was higher in the SIRD-RII subgroup but lower in the MARD-II subgroup. The pathophysiological mechanisms underlying the contrasting pattern of LPC between these two subgroups remain unknown.

In agreement with previous studies, the clinical variable-derived subgroups also demonstrated distinct cardio-renal risks in our cohort [1]. The SIRD-RII subgroup shows the highest cardio-renal risks, as manifested by a significantly higher risk for heart failure and progressive CKD. Our finding is consistent with data from the ANDIS cohort, the German Diabetes Study and a retrospective study in a Japanese population, which showed that the SIRD subgroup was at increased risk for progressive CKD [8, 11, 12]. We extended these previous studies by showing that heart failure may be another important adverse clinical outcome associated with SIRD that warrants further studies. The excessive cardio-renal risk in the SIRD-RII subgroup is attributable to the more severe clinical risk factors, including poor glycaemic control and dyslipidaemia, as well as novel risk factors such as activation of the ceramide pathway and increased inflammation tone [40]. Interestingly, we also observed that the MARD-II subgroup had a moderately elevated risk for progressive CKD. The mean age in this subgroup is only 4 years older than that in the MOD subgroup. Hence, ageing is unlikely to account for the difference in CKD risk. It is possible that diabetes may remain undiagnosed for a longer period in the MARD-II subgroup due to the less pronounced metabolic risk profile, but this is speculative.

Our study adds evidence to support that data-driven cluster analysis upon clinical variables may set the foundation for precision medicine. In addition to diabetes prevention and healthcare resource allocation, it may have implications for precision of medication treatment. The MARD-II subgroup has a low insulin secretion and high PRS for beta cell dysfunction. Patients in this subgroup may better respond to insulin secretagogues, as shown in the ADOPT trial [21]. On the other hand, patients in the SIRD-RII and MOD subgroups may respond better to medications that improve insulin sensitivity beyond and above interventions for weight loss. The SIRD-RII subgroup may benefit from early administration of sodium–glucose cotransporter 2 inhibitors and glucagon-like peptide 1 receptor agonists, given the high cardio-renal risk in this subgroup [41].

The strengths of the current study include a well-characterised cohort with a long follow-up. We included only participants with recent-onset type 2 diabetes to partly mitigate confounding, and used the same clustering algorithm on the same clinical variables to enable reasonable comparison between this Asian study population and patients of European descent. Nevertheless, several important weaknesses must be highlighted. First, the sample size is moderate, and thus our study on the insulin resistance PRS may be underpowered. Second, although we have excluded type 1 diabetes on the basis of clinical criteria, we did not measure GAD antibody. As shown in our analyses using the type 1 diabetes PRS, we cannot exclude the possibility that a small proportion of participants may have autoimmune-related diabetes. Third, we used the triacylglycerol/HDL-cholesterol ratio instead of HOMA indices to cluster the validation cohort for the lipidomics study. Although this may be considered a reasonable approach, as shown by a comparable cardio-renal risk profile (ESM Fig. 6), the concordance of subgroup assignments derived from two sets of classifying variables is moderate (Cohen’s kappa 0.72, ESM Table 11), especially for the MOD and MARD-II subgroups. Finally, we have not performed an external validation. Hence, the generalisability of our findings should be assessed in future studies.

In summary, cluster analysis identified three subgroups of patients with recent-onset type 2 diabetes in our South East Asian cohort. These subgroups demonstrate not only distinct clinical phenotypes but also differences in genetic aetiology, pathophysiology features and cardio-renal risks. Together with previous studies in other populations, our data suggest that cluster analysis on clinically available variables may be used as a starting point to stratify the heterogeneous diabetic population into subgroups for precision medicine.

## Supplementary information

ESM (PDF 1238 kb)

## Data Availability

The datasets generated and analysed during the current study are not publicly available. However, anonymised data are available from the corresponding author upon reasonable request.

## Abbreviations

ANDIS: All New Diabetics in Scania
CKD: Chronic kidney disease
GWAS: Genome-wide association study
LPC: Lysophosphatidylcholine
MACE: Major adverse cardiovascular events
MARD-II: Mild age-related diabetes with insulin insufficiency
MOD: Mild obesity-related diabetes
PRS: Polygenic risk score
SIDD: Severe insulin-deficient diabetes
SIRD-RII: Severe insulin-resistant diabetes with relative insulin insufficiency
