# Correction: Clinical variable-based cluster analysis identifies novel subgroups with a distinct genetic signature, lipidomic pattern and cardio-renal risks in Asian patients with recent-onset type 2 diabetes

**DOI:** 10.1007/s00125-022-05783-6

**Published:** 2022-09-07

**Authors:** Jiexun Wang, Jian-Jun Liu, Resham L. Gurung, Sylvia Liu, Janus Lee, M. Yiamunaa, Keven Ang, Yi Ming Shao, Justin I-Shing Tang, Peter I. Benke, Federico Torta, Markus R. Wenk, Subramaniam Tavintharan, Wern Ee Tang, Chee Fang Sum, Su Chi Lim

**Affiliations:** 1 Clinical Research Unit, Khoo Teck Puat Hospital, Singapore, Republic of Singapore; 2 Department of Medicine, Khoo Teck Puat Hospital, Singapore, Republic of Singapore; 3 Lipidomics Incubator, Yong Loo Lin School of Medicine, National University of Singapore, Singapore, Republic of Singapore; 4 Diabetes Centre, Admiralty Medical Centre, Singapore, Republic of Singapore; 5 National Healthcare Group Polyclinic, Singapore, Republic of Singapore; 6 Saw Swee Hock School of Public Health, National University of Singapore, Singapore, Republic of Singapore; 7 Lee Kong Chian School of Medicine, Nanyang Technological University, Singapore, Republic of Singapore

**Correction: Diabetologia**

10.1007/s00125-022-05741-2

The article “Clinical variable-based cluster analysis identifies novel subgroups with a distinct genetic signature, lipidomic pattern and cardio-renal risks in Asian patients with recent-onset type 2 diabetes”, written by Jiexun Wang, Jian-Jun Liu, Resham L. Gurung, Sylvia Liu, Janus Lee, Yiamunaa M, Keven Ang, Yi Ming Shao, Justin I-Shing Tang, Peter I. Benke, Federico Torta, Markus R. Wenk, Subramaniam Tavintharan, Wern Ee Tang, Chee Fang Sum, Su Chi Lim, was originally published electronically on the publisher’s internet portal on 28 June 2022 without open access. With the author(s)’ decision to opt for Open Choice the copyright of the article changed on 9 August 2022 to © The Author(s) 2022 and the article is forthwith distributed under a Creative Commons Attribution 4.0 International License, which permits use, sharing, adaptation, distribution and reproduction in any medium or format, as long as you give appropriate credit to the original author(s) and the source, provide a link to the Creative Commons licence, and indicate if changes were made. The images or other third party material in this article are included in the article's Creative Commons licence, unless indicated otherwise in a credit line to the material. If material is not included in the article's Creative Commons licence and your intended use is not permitted by statutory regulation or exceeds the permitted use, you will need to obtain permission directly from the copyright holder. To view a copy of this licence, visit http://creativecommons.org/licenses/by/4.0/.

