# Supplementary material for: Clinical variable-based cluster analysis identifies novel subgroups with a distinct genetic signature, lipidomic pattern and cardio-renal risks in Asian patients with recent-onset type 2 diabetes
Source: Diabetologia. 2022 Jun 28;65(12):2146–56. doi: 10.1007/s00125-022-05741-2 (PMC9630229; doi:10.1007/s00125-022-05741-2)
Supplement: Supplementary file 1 — (PDF 1238 kb) [file 125_2022_5741_MOESM1_ESM.pdf]

Clinical variable- based cluster analysis identifies novel subgroups with a distinct genetic signature, lipidomic pattern and cardio-renal risks in Asian patients with recent-onset type 2 diabetes

Jiexun Wang, Jian-Jun Liu, Resham L Gurung, Sylvia Liu, Janus Lee, Yiamunaa M, Keven Ang, Yi Ming Shao, Justin I-Shing Tang, Perter I Benke, Federico Torta, Markus R Wenk, Subramaniam Tavintharan, Wern Ee Tang, Chee Fang Sum and Su Chi Lim

ESM Methods

Beta cell dysfunction, insulin resistance and type 1 diabetes polygenic risk score (PRS)

Beta cell dysfunction PRS was created based on 35 single nucleotide polymorphisms (SNPs) associated with insulin secretion in Asian population. We weighted the SNPs by their effect on risk of type 2 diabetes in the East Asian population to study whether the novel subgroups differ in genetic risks for development of type 2 diabetes [1]. Specifically, we assumed an additive genetic model and applied a linear weighting of 0, 1, and 2 for genotypes containing 0, 1, and 2 risk alleles, respectively, i.e. weighted PRS = (β1× SNP1 + β2× SNP2 + … + βn× SNPn) × (n/sum of the β-coefficients), where SNPi is the number of risk allele for each SNP and βi is the effect size for that specific SNP. We created insulin resistance PRS based on 20 SNPs associated with insulin sensitivity by the same approach. A high PRS score indicates a high genetic risk for beta cell dysfunction and insulin resistance, respectively. Assuming a log- additive model, we included 9 SNPs associated with risk for type 1 diabetes in non-Caucasian population to

construct PRS for type 1 diabetes. For the 7 non-DR3/DR4 SNPs, we applied a linear weighting of 0, 1, and 2 for genotypes containing 0, 1, and 2 risk alleles and multiplied by their effects on risk of type 1 diabetes. For DR3/DR4-DQ8 contribution, we imputed DR3/DR4-DQ8 haplotypes and the corresponding weights were assigned to each individuals score [2-4]. The final type 1 diabetes PRS was obtained from the sum of these two sets of SNPs divided by 15. GWAS genotyping, quality control procedures and principal component (PC) analysis have been described previously [5].

Lipidomics Assay by LC-MS

Plasma samples were randomized for each cohort, respectively, before analytical assay. Batch quality control (BQC) samples were prepared by pooling equal amount of aliquot from all plasma samples in each cohort, respectively, before lipid extraction. Plasma (10 µL) was mixed with 190 µL 1-butanol/methanol (BuMe, 1:1 v/v) containing internal standards. The mixture was vortexed for 30 s, then sonicated for 30 min at 20°C. The samples were then centrifuged at 14,000 x g for 10 min at 10°C and the supernatant was carefully transferred into autosampler vials, not to disturb the precipitated protein pellets. Extracted blanks were prepared using the same extraction protocol, using 10 µl BuMe instead of plasma samples. Technical quality control (TQC) samples were generated by pooling the lipid extracts of study samples to measure instrumental variability.

Reverse Phase chromatographic separation of plasma samples was based on a modified version of Huynh et al [6]. The analysis was carried out on an Agilent 6495 QQQ and Infinity-II LC-MS system, using a Zorbax RRHD Eclipse Plus C18, 95Å (2.1 x 100 mm, 1.8 µm) column. The mobile

phases consisted of (A) 10 mmol/L ammonium formate and formic acid (0.1%) in water/acetonitrile/2-propanol (50:30:20, v/v) and (B) 10 mmol/L ammonium formate and formic acid (0.1%) in 2-propanol/ acetonitrile/water (90:9:1, v/v). Using a flow rate of 0.4 mL/min, the gradient started from 15% B to 50% B in 2.5 min, 50 to 57% B in 0.1 min, 57% B to 70% B from 2.6 to 9 min, 70% B to 93% B from in 0.1 min, 93% B to 96% B from 9.1 to 11 min, 96% B to 100% B in 0.1 min, where it was maintained till 11.9 min, then re-equilibrated at 15% B for 3 min prior to the next injection. The injection volume was 2 µL. Autosampler and column thermostat temperature were at 15°C and 45°C, respectively. Total method run time, including needle wash, was 16.1 min. To test the linear response, TQCs extracts were injected at different volumes.

The mass spectrometer conditions were as follows: Capillary voltage 3500V, Drying gas temperature and flow rate 150°C and 17L/min, Sheath gas temperature and flow rate 200°C and 10L/min, Nebulizer pressure 20 psi. Targeted analysis was performed in Dynamic MRM positive ion mode, using “unit” resolution (0.7 amu) for Q1 and Q3 isolation width.

Chromatographic peaks were annotated based on retention time and specific MRM transitions using Agilent MassHunter Quantitative Analysis software (version B.10.1). Internal standards were used to normalize the raw peak areas in the corresponding lipid class. Endogenous species were quantified using one standard per lipid class thus our method can only deliver relative quantitation results.

References:

[1] Spracklen CN, Horikoshi M, Kim YJ, et al. (2020) Identification of type 2 diabetes loci in 433,540 East Asian individuals. Nature 582(7811): 240-245. 10.1038/s41586-020-2263-3

[2] Harrison JW, Tallapragada DSP, Baptist A, et al. (2020) Type 1 diabetes genetic risk score is discriminative of diabetes in non-Europeans: evidence from a study in India. Sci Rep 10(1): 9450. 10.1038/s41598-020-65317-1

[3] Oram RA, Patel K, Hill A, et al. (2016) A Type 1 Diabetes Genetic Risk Score Can Aid Discrimination Between Type 1 and Type 2 Diabetes in Young Adults. Diabetes Care 39(3): 337-344. 10.2337/dc15-1111

[4] Prasad RB, Asplund O, Shukla SR, et al. (2022) Subgroups of patients with young-onset type 2 diabetes in India reveal insulin deficiency as a major driver. Diabetologia 65(1): 65-78. 10.1007/s00125-021-05543-y

[5] Lim SC, Dorajoo R, Zhang X, et al. (2017) Genetic variants in the receptor for advanced glycation end products (RAGE) gene were associated with circulating soluble RAGE level but not with renal function among Asians with type 2 diabetes: a genome-wide association study. Nephrol Dial Transplant 32(10): 1697-1704. 10.1093/ndt/gfw263

[6] Huynh K, Barlow CK, Jayawardana KS, et al. (2019) High-Throughput Plasma Lipidomics: Detailed Mapping of the Associations with Cardiometabolic Risk Factors. Cell Chem Biol 26(1): 71-84 e74. 10.1016/j.chembiol.2018.10.008

ESM Table 1: Single nucleotide polymorphisms (SNPs) and weights for construction of β cell dysfunction and insulin resistance polygenic risk scores (PRS)

SNP	Nearest gene	R/A	Effect size
**Beta cell dysfunction**			
rs340874	PROX1	C/T	0.0435
rs11717959	IGF2BP2	G/T	-0.0937
rs4481184	IGF2BP2	T/C	0.1198
rs6780171	IGF2BP2	A/T	0.1209
rs1801212	WFS1	A/G	0.1711
rs4457053	ZBED3	G/A	0.0736
rs7756992	CDKAL1	G/A	0.1616
rs9379084	RREB1	G/A	0.0718
rs9505097	RREB1	C/T	0.0443
rs10228066	DGKB	T/C	0.0646
rs791595	LEP	A/G	0.1165
rs878521	GCK	A/G	0.0288
rs13262861	ANK1	C/A	0.1000
rs3802177	SLC30A8	G/A	0.1104
rs4736819	ANK1	T/C	0.0720
rs10757283	CDKN2A/B	T/C	-0.0564
rs10811660	CDKN2A/B	G/A	0.1822
rs10974438	GLIS3	C/A	0.0670
rs12378717	GPSM1	G/C	0.1248
rs505922	ABO	C/T	0.0424
rs10882101	HHEX/IDE	T/C	0.1273
rs1112718	HHEX/IDE	A/G	0.1410
rs11257655	CDC123/CAMK1D	T/C	0.1140
rs7903146	TCF7L2	T/C	0.2724
rs102275	TMEM258	T/C	0.0295
rs10830963	MTNR1B	G/C	0.0372
rs2237895	KCNQ1	C/A	0.1982
rs2237897	KCNQ1	C/T	0.2476
rs234853	KCNQ1	G/A	-0.1805
rs445084	KCNQ1	G/A	0.0518
rs77464186	CENTD2/ARAP1	A/C	0.1399
rs10842994	KLHDC5	C/T	0.0553
rs1359790	SPRY2	G/A	0.0877
rs1005752	HMG20A	A/C	0.0701
rs8038040	C2CD4A/B	G/A	0.0795

Insulin resistance			
rs3768321	MACF1	T/G	0.0572
rs10195252	GRB14/COBLL1	T/C	0.0552
rs1260326	GCKR	C/T	0.0632
rs2052261	CEP68	G/A	-0.0272
rs2249105	CEP68	A/G	0.0293
rs2972144	IRS1	G/A	0.0464
rs11709077	PPARG	G/A	0.1097
rs11926707	KIF9	C/T	0.0157
rs28819812	PDGFC	C/A	0.0153
rs465002	ANKRD55	T/C	0.0734
rs702634	ARL15	A/G	0.0470
rs9687832	ANKRD55	A/G	0.0410
rs2280141	PLEKHA1	T/G	0.0433
rs2738809	BCAR1	G/A	0.0185
rs2925979	CMIP	T/C	0.0423
rs12454712	BCL2A	T/C	0.0543
rs10406431	GIPR	A/G	0.0893
rs2238689	GIPR	C/T	0.0723
rs8107974	TM6SF2	T/A	0.0282
rs889138	PEPD	C/T	0.0552

R, risk allele; A, alternative allele.

Weight (effect size) for each SNP was derived from meta-analysis of GWAS data from 77,418 individuals with T2D and 356,122 healthy individuals of East Asian ancestry (PMID: 32499647) and calculated as natural log (Odds Ratio). Please also refer to ESM methods.

ESM Table 2: Single nucleotide polymorphisms (SNPs) for construction of type 1 diabetes polygenic risk scores (PRS)

<table>
<tr><th>SNP</th><th>Risk allele</th><th>Gene</th><th>Odds Ratio</th><th>Weight</th></tr>
<tr><td>rs2476601</td><td>A</td><td>PTPN22</td><td>1.96</td><td>0.67</td></tr>
<tr><td>rs1264813</td><td>T</td><td>HLA-A*24</td><td>1.54</td><td>0.43</td></tr>
<tr><td>rs2395029</td><td>T</td><td>HLA-B*5701</td><td>2.5</td><td>0.92</td></tr>
<tr><td>rs3129889</td><td>A</td><td>HLA-DRB1*15</td><td>14.88</td><td>2.70</td></tr>
<tr><td>rs12722495</td><td>T</td><td>IL2RA</td><td>1.58</td><td>0.46</td></tr>
<tr><td>rs689</td><td>T</td><td>INS</td><td>1.75</td><td>0.56</td></tr>
<tr><td>rs2292239</td><td>T</td><td>ERBB3</td><td>1.35</td><td>0.30</td></tr>
<tr><td rowspan="5">rs2187668
rs7454108</td><td rowspan="5">T
C</td><td>DR3/DR4-DQ8</td><td>48.18</td><td>3.87</td></tr>
<tr><td>DR3/DR3</td><td>21.12</td><td>3.05</td></tr>
<tr><td>DR4-DQ8/DR4-DQ8</td><td>21.98</td><td>3.09</td></tr>
<tr><td>DR4-DQ8/X</td><td>7.03</td><td>1.95</td></tr>
<tr><td>DR3/X</td><td>4.53</td><td>1.51</td></tr>
</table>

Details on type 1 diabetes PRS calculation have been described in ESM methods

ESM Table 3: participant baseline characteristics in lipidomics study

Variable	Discovery	Validation
Number of participants	687	226
Type 2 diabetes subgroups, N (%)		
SIRD-RII	130 (18.9)	49 (21.7)
MOD	307 (44.7)	95 (42.0)
MARD-II	250 (36.4)	82 (36.3)
Index age, years	53.8 (11.6)	50.0 (10.2)
Diabetes onset age, years	51.0 (11.5)	48.4 (10.9)
Male sex, N (%)	349 (50.8)	139 (61.5)
Ethnicity, N (%)		
Chinese	328 (48.7)	143 (63.3)
Malay	155 (23.0)	53 (23.5)
Asian Indian	190 (28.2)	30 (13.3)
Diabetes duration, years	3.0 [1.0, 5.0]	3.0 [1.0, 4.0]
Body mass index, kg/m^2	28.5 (5.4)	27.8 (5.8)
HbA1c, %	7.4 (1.3)	8.1 (1.9)
Blood pressure, mmHg		
Systolic	137 (17)	133 (17)
Diastolic	80 (9.0)	80 (11)
Kidney function		
eGFR, $ml/min/1.73m^2$	95 (21)	94 (25)
Urine ACR, ug/mg	13.0 [4.0, 42.0]	19.0 [8.0, 71.5]
Clinical lipid, mmol/L		
HDL cholesterol	1.30 (0.38)	1.27 (0.37)
LDL cholesterol	2.90 (0.85)	3.01 (0.90)
Triacylglycerol	1.38 [1.05, 1.92]	1.62 [1.12, 2.36]
Triacylglycerol/HDL ratio	1.1 [0.8, 1.7]	1.4 [0.8, 2.1]
Medication usage, N (%)		
Insulin	64 (9.4)	35 (16.6)
Statins	507 (74.1)	159 (70.7)
RAS blockers	322 (47.3)	111 (49.3)

ACR, albumin-to-creatinine ratio; RAS, renin-angiotensin system; MOD, mild obesity- related diabetes; SIRD-RII, severe insulin- resistant diabetes with relative insulin insufficiency; MARD-II, mild age- related diabetes with insulin insufficiency

ESM Table 4: Participant baseline characteristics in lipidomics validation study stratified by three subgroups (N=226)

Variables	MOD	SIRD-RII	MARD-II	p value
Number of Participants (%)	95 (42.0)	49 (21.7)	82 (36.3)	
Age, years	50.0 [40.0, 61.0]	41.0 [33.0, 50.0]	56.0 [50.0, 63.0]	<0.001
Diabetes onset age, years	47.0 [38.0, 56.5]	38.0 [30.8, 47.0]	52.5 [47.2, 61.0]	<0.001
Male sex, N(%)	66 (69.5)	30 (61.2)	43 (52.4)	0.07
Ethnicity, N(%)				0.02
Chinese	56 (58.9)	25 (51.0)	62 (75.6)	
Malay	25 (26.3)	18 (36.7)	10 (12.2)	
Asian Indian	14 (14.7)	6 (12.2)	10 (12.2)	
Diabetes duration, years	2.0 [1.0, 4.0]	3.0 [1.0, 4.0]	3.0 [1.3, 4.0]	0.66
Body mass index, kg/m^2	30.4 (4.6)	30.8 (6.6)	22.9 (2.6)	<0.001
HbA1c, %	7.1 (0.9)	10.5 (1.8)	7.8 (1.5)	<0.001
Blood pressure, mmHg				
Systolic	137 (16)	130 (15)	130 (19)	0.005
Diastolic	82 (11)	81 (12)	77 (10)	0.003
Kidney function				
eGFR, ml/min/1.73m^2	92 (24)	104 (22)	89 (25)	0.002
Urine ACR, ug/mg	19.0 [8.3, 91.0]	23.0 [8.0, 70.3]	17.0 [7.0, 36.0]	0.65
Clinical lipid, mmol/L				
HDL cholesterol	1.19 (0.29)	1.07 (0.30)	1.49 (0.39)	<0.001
LDL cholesterol	2.94 (0.79)	3.19 (1.13)	2.98 (0.85)	0.38
Triacylglycerol	1.73 [1.33, 2.37]	2.41 [1.84, 3.85]	1.13 [0.86, 1.61]	<0.001
Triacylglycerol/ HDL ratio	1.51 [1.11, 2.12]	3.01 [1.68, 4.08]	0.80 [0.59, 1.18]	<0.001
Medication usage, N (%)				
Insulin	9 (9.9)	18 (39.1)	8 (10.8)	<0.001
Statins	68 (71.6)	32 (66.7)	59 (72.0)	0.79
RAS blockers	55 (57.9)	22 (45.8)	34 (41.5)	0.08

ACR, albumin-to-creatinine ratio; RAS, renin-angiotensin system; MOD, mild obesity- related diabetes; SIRD-RII, severe insulin- resistant diabetes with relative insulin insufficiency; MARD-II, mild age- related diabetes with insulin insufficiency

ESM Table 5: Lipid species differed significantly between SIRD-RII and MOD subgroups

	Discovery		Validation	
**Lipid species**	**Coefficient #**	***p* value ##**	**Coefficient #**	***p* value ###**
PC (O-36:1)	0.17	3.17E-05	0.18	1.17E-02
PC (O-36:4)	0.19	3.21E-06	0.23	1.28E-03
PC (O-38:4)	0.20	1.24E-06	0.28	1.22E-04
PC (P-40:5)	0.14	8.01E-04	0.19	8.82E-03
PE (34:1)	0.13	1.04E-03	0.30	1.06E-05
PE (36:4)	0.14	9.83E-04	0.34	1.48E-06
PE (P-16:0/22:5)	0.20	2.02E-06	0.25	6.06E-04
PE (P-18:0/18:1)	0.16	6.93E-05	0.27	1.58E-04
PE (P-18:1/20:4)	0.16	6.87E-05	0.20	4.68E-03
Cer (d18:1/18:0)	0.22	6.06E-08	0.24	6.73E-04
Cer (d18:1/22:0)	0.21	5.33E-07	0.24	6.14E-04
Cer (d18:1/23:0)	0.17	5.66E-05	0.19	8.75E-03
SM (36:0)	0.20	2.27E-07	0.25	2.64E-04
SM (38:0)	0.19	1.28E-06	0.19	3.17E-03
SM (40:1)	0.19	4.71E-06	0.19	6.89E-03
SM (41:0)	0.20	5.02E-07	0.27	1.12E-04
SM (41:3)	0.22	1.51E-07	0.27	2.13E-04

positive coefficient indicates lipid levels were higher in SIRD-RII as compared to MOD subgroup.
p value < 0.0011 was considered statistically significant in discovery cohort based on Bonferroni correction threshold (0.05/45=0.0011)
lipids in validation cohort with nominal p <0.05 and having the same coefficient direction as that in discovery cohort were considered as statistically significant.
PE, phosphatidylethanolamine; PC, phosphatidylcholine; Cer, ceramide; SM, sphingomyelin; MOD, mild obesity- related diabetes; SIRD-RII, severe insulin- resistant diabetes with relative insulin insufficiency

ESM Table 6: Lipid species differed significantly between MARD-II and MOD subgroups

	Discovery		Validation	
**Lipid species**	**Coefficient #**	***p* value ##**	**Coefficient #**	***p* value ###**
PC (32:1)	-0.16	5.87E-05	-0.26	1.27E-04
PC (38:3)	-0.23	1.89E-08	-0.32	6.51E-06
PC (O-36:1)	0.17	3.95E-05	0.23	1.72E-03
PC (O-36:2)	0.21	5.56E-07	0.21	3.36E-03
PC (P-36:2)	0.20	1.51E-06	0.32	6.27E-06
PC (P-40:5)	0.16	6.74E-05	0.25	5.18E-04
PC (P-40:6)	0.15	1.96E-04	0.27	1.43E-04
LPC (19:0)	0.18	1.29E-05	0.15	3.56E-02
LPC (19:1)	0.22	4.18E-08	0.20	5.65E-03
LPC (O-22:0)	0.22	1.38E-07	0.15	4.32E-02
LPC (O-22:1)	0.19	2.10E-06	0.14	5.00E-02
LPC (O-24:0)	0.20	1.11E-06	0.23	1.66E-03
LPC (O-24:1)	0.21	1.96E-07	0.14	4.29E-02
LPC (O-24:2)	0.27	1.52E-11	0.19	8.21E-03
PE (36:1)	-0.19	1.59E-06	-0.18	7.38E-03
PE (38:3)	-0.17	4.73E-05	-0.20	5.37E-03
PE (40:6)	-0.16	9.28E-05	-0.14	4.36E-02
PI (32:1)	-0.19	3.40E-06	-0.27	1.01E-04
Hex1Cer(d18:1/24:0)	0.20	1.69E-06	0.21	4.05E-03
SM (36:0)	-0.23	3.63E-09	-0.26	1.31E-04
SM (38:0)	-0.20	5.81E-07	-0.35	1.54E-07
SM (41:0)	-0.13	8.15E-04	-0.19	5.22E-03
SM (44:3)	0.15	2.65E-04	0.28	4.85E-05

a positive coefficient indicates lipid levels were higher in MARD-II as compared to MOD subgroup. Vice versa, a negative coefficient indicates lipid levels were lower in MARD-II as compared to MOD subgroup.

p value < 0.0011 was considered statistically significant in discovery cohort based on Bonferroni correction threshold 0.05/45=0.0011

lipids in validation cohort with a nominal p <0.05 and having the same coefficient direction as that in discovery cohort were considered statistically significant.

PC, phosphatidylcholine; LPC, lysophosphatidylcholine; PE, phosphatidylethanolamine; Cer, ceramide; SM, sphingomyelin; MOD, mild obesity- related diabetes; MARD-II, mild age- related diabetes with insulin insufficiency

ESM Table 7: Adverse clinical outcomes in three novel subgroups

Outcome	Overall	MOD	SIRD-RII	MARD-II
**Progressive CKD #**				
Number of participants	554	246	113	195
Cases/Person-years	41/3853	12/1757	12/755	17/1342
Incidence rate ## (95% CI)	10.6 (7.6-14.4)	6.8 (3.5-11.9)	15.9 (8.2 - 27.8)	12.7 (7.4-20.3)
**Incident Heart failure**				
Number of participants	687	307	130	250
Cases/person-years	38/4850	14/2201	13/897	11/1753
Incidence rate# (95% CI)	7.8 (5.5-10.8)	6.4 (3.5-10.7)	14.5 (7.7-24.8)	6.3 (3.1-11.2)
**MACE**				
Number of participants	687	307	130	250
Cases/person-years	35/4764	18/2136	7/910	10/1719
Incidence rate ## (95% CI)	7.3 (5.1-10.2)	8.4 (5.0-13.3)	7.7 (3.1-15.9)	5.8 (2.8-10.7)
**Stroke ###**				
Number of participants	687	307	130	250
Cases/person-years	14/4782	6/2150	3/910	5/1723
Incidence rate ## (95% CI)	2.9 (1.6-4.9)	2.8 (1.0-6.1)	3.3 (0.7-9.6)	2.9 (0.9-6.8)
**AMI ###**				
Number of participants	687	307	130	250
Cases/person-years	12/4790	9/2146	2/918	1/1727
Incidence rate ## (95% CI)	2.5 (1.3-4.4)	4.2 (1.9-8.0)	2.2 (0.3-7.9)	0.6 (0.02-3.2)
**All-cause mortality**				
Number of participants	687	307	130	250
Cases/person-years	40/4814	17/2167	8/918	15/1730
Incidence rate ## (95% CI)	8.3 (5.9-11.3)	7.9 (4.6-12.6)	8.7 (3.8-17.2)	8.7 (4.8-14.3)

CKD, chronic kidney disease, MACE, major adverse cardiovascular events; AMI, acute myocardial infarcction

Only participants with baseline eGFR above 60 ml/min/1.73m^2 were included in the analysis on progressive CKD

Incidence rates were presented as events per 1,000 person-years.

stroke and AMI events were not analysed separately due to small event numbers.

MOD, mild obesity- related diabetes; SIRD-RII, severe insulin- resistant diabetes with relative insulin insufficiency; MARD-II, mild age- related diabetes with insulin insufficiency

ESM Table 8: Effect of sex on cluster analysis by k-means

	New clustering after adjustment for sex			
**Original clustering**	**MOD**	**SIRD-RII**	**MARD-II**	**Total**
MOD	289 (94.1%)	13 (4.2%)	5 (1.6%)	307 (100%)
SIDD- RII	6 (4.6%)	113 (86.9%)	11 (8.5%)	130 (100%)
MARD-II	15 (6.0%)	8 (3.2%)	227 (91.8%)	250 (100%)

MOD, mild obesity- related diabetes; SIRD-RII, severe insulin- resistant diabetes with relative insulin insufficiency; MARD-II, mild age- related diabetes with insulin insufficiency

ESM Table 9: Risks for adverse clinical outcomes in novel subgroups derived from ANDIS cohort centroids

	**SIDD**	**SIRD**	**MOD**	**MARD**
**Progressive CKD #**				
Number of participants	94	108	127	225
Cases/Person-years	10/628.0	6/770.2	9/880.3	16/1573.6
Incidence rate ## (95% CI)	15.9 (7.6-29.2)	7.8 (2.9-17.0)	10.2 (4.7-19.4)	10.2 (5.8-16.5)
**Heart failure**				
Number of participants	115	136	151	285
Cases/person-years	10/796.1	7/974.8	7/1076.1	14/2003.2
Incidence rate ## (95% CI)	12.5 (6.0-23.1)	7.2 (2.9-14.8)	6.5 (2.6-13.4)	7.0 (3.8-11.7)
**MACE**				
Number of participants	115	136	151	285
Cases/person-years	7/807.6	8/950.5	5/1058.5	15/1947.0
Incidence rate ## (95% CI)	8.7 (3.5-17.9)	8.4 (3.6-16.6)	4.7 (1.5-11.0)	7.7 (4.3-12.7)
**All-cause mortality**				
Number of participants	115	136	151	285
Cases/person-years	8/816.4	11/960.7	4/1066.7	17/1971.0
Incidence rate ## (95% CI)	9.8 (4.2-19.3)	11.1 (5.7-20.5)	3.7 (1.0-9.6)	8.6 (5.0-13.8)

Only participants with baseline eGFR above 60 ml/min/1.73m^2 were included in the analysis on progressive CKD

Incidence rates were presented as event number per 1,000 person-years.

SIDD, severe insulin- deficient diabetes; SIRD, severe insulin- resistant diabetes; MOD, mild obesity-related diabetes; MARD, mild age-related diabetes

ESM Table 10: Differences in fasting plasma glucose, c-peptide and HOMA indices between participants with type 1 diabetes (T1D) PRS score in top 5 percentiles and the remaining 95 percentiles

	Participants with T1D PRS ≥ 95th percentile	Patients with T1D PRS < 95th percentile	*p* value
Number of participants	38	648	
Novel subgroups, N (%)			0.02
MOD	9 (2.9)	298 (97.1)	
SIRD-RII	12 (9.2)	118 (91.8)	
MARD-II	17 (6.8)	232 (93.2)	
Fasting plasma glucose, mmol/L	8.2 (2.5)	7.6 ± 2.3	0.14
C-peptide, pmol/L	709 [541, 857]	790 [560, 1062]	0.19
HOMA2-B, %	57 [46, 74]	69 [49, 97]	0.02
HOMA2-IR	1.7 [1.3, 2.2]	1.9 [1.3, 2.7]	0.39

T1D, type 1 diabetes; MOD, mild obesity- related diabetes; SIRD-RII, severe insulin- resistant diabetes with relative insulin insufficiency; MARD-II, mild age- related diabetes with insulin insufficiency

EMS Table 11: Concordance between clusters derived from classifiers with HOMA indices included and that by replacing HOMA indices with TG/HDL cholesterol ratio

		Cluster Analysis Using Alternative Variables		
		**SIRD-RII**	**MARD-II**	**MOD**
**Original Cluster Analysis (onset age, BMI, HbA1c, HOMA2-B and HOMA2-IR)**	**SIRD-RII**	123 (94.6%)	6 (4.6%)	1 (1%)
	**MARD-II**	21 (8.4%)	217 (87.1%)	11 (4.4%)
	**MOD**	6 (2.0%)	80 (26.0%)	221 (72.0%)

Cohen’s kappa =0.72

MOD, mild obesity- related diabetes; SIRD-RII, severe insulin- resistant diabetes with relative insulin insufficiency; MARD-II, mild age- related diabetes with insulin insufficiency

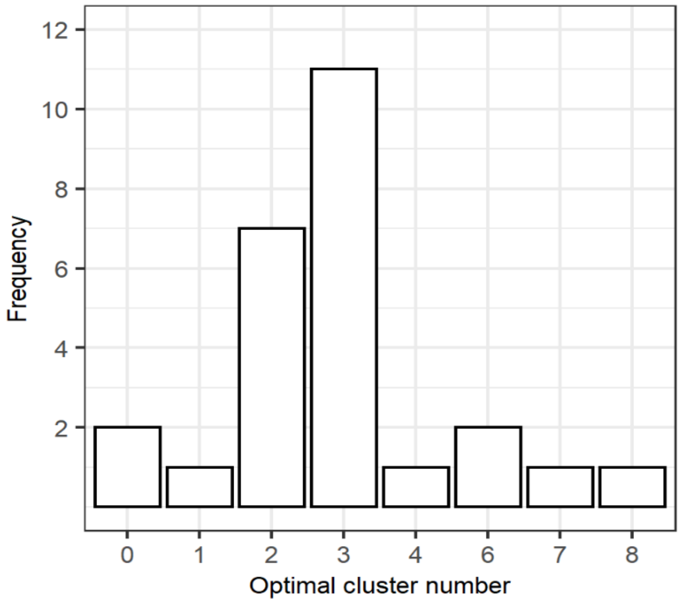

ESM Figure 1: Determination of optimal cluster number by majority voting from 26 metrics using R package "NbClust"

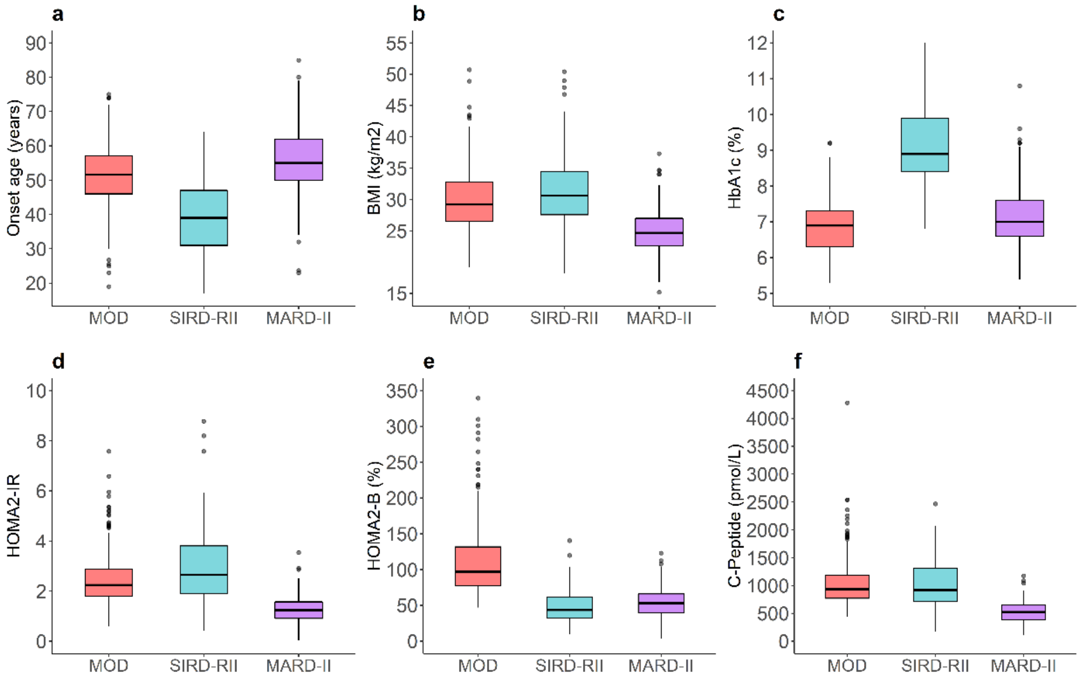

ESM Figure 2: Levels of clinical classifiers in three novel subgroups

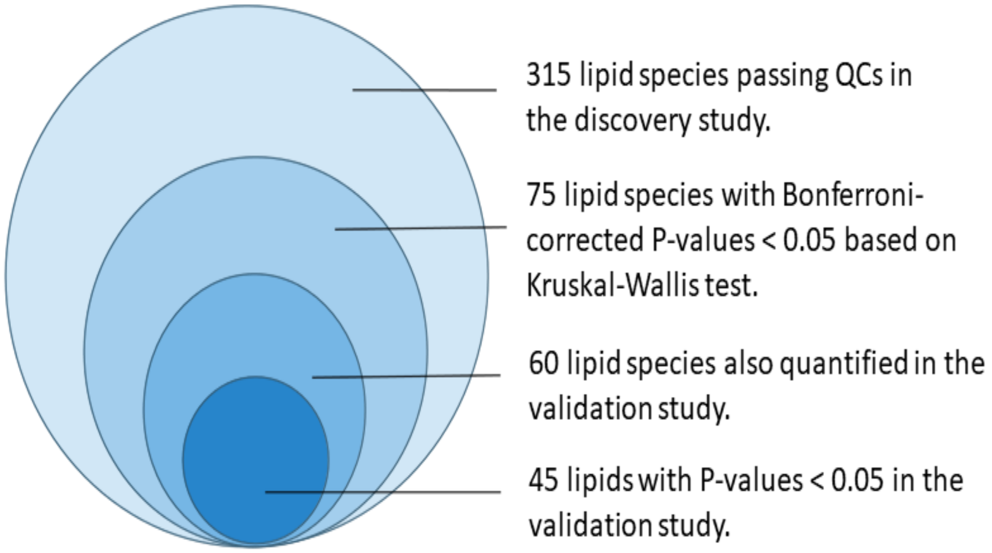

ESM Figure 3: procedure of statistical analysis on lipidomics data

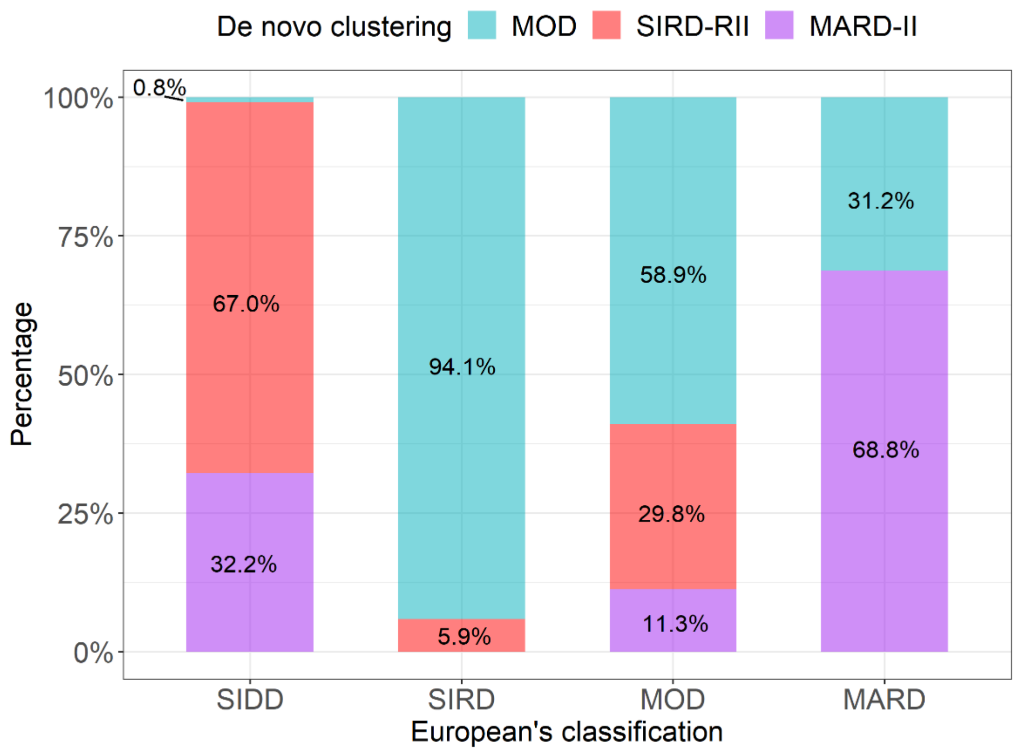

ESM Figure 4: Overlapping between clusters derived from centroids of ANDIS cohort (*Ahlqvist et al*. Lancet Diabetes and Endocrinology 2018;6:361) and those from de novo clustering

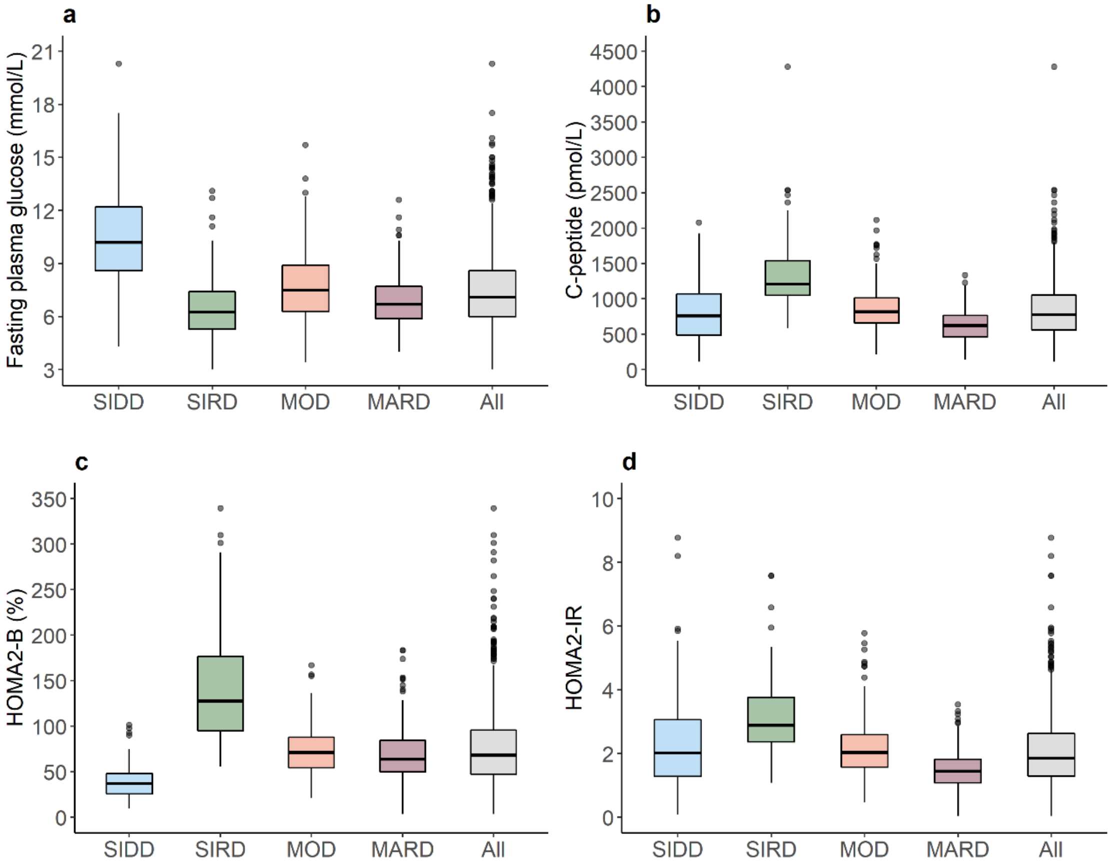

ESM Figure 5: Fasting glucose, C-peptide, HOMA2-B and HOMA2-IR levels in novel subgroups derived from ANDIS cohort centroids

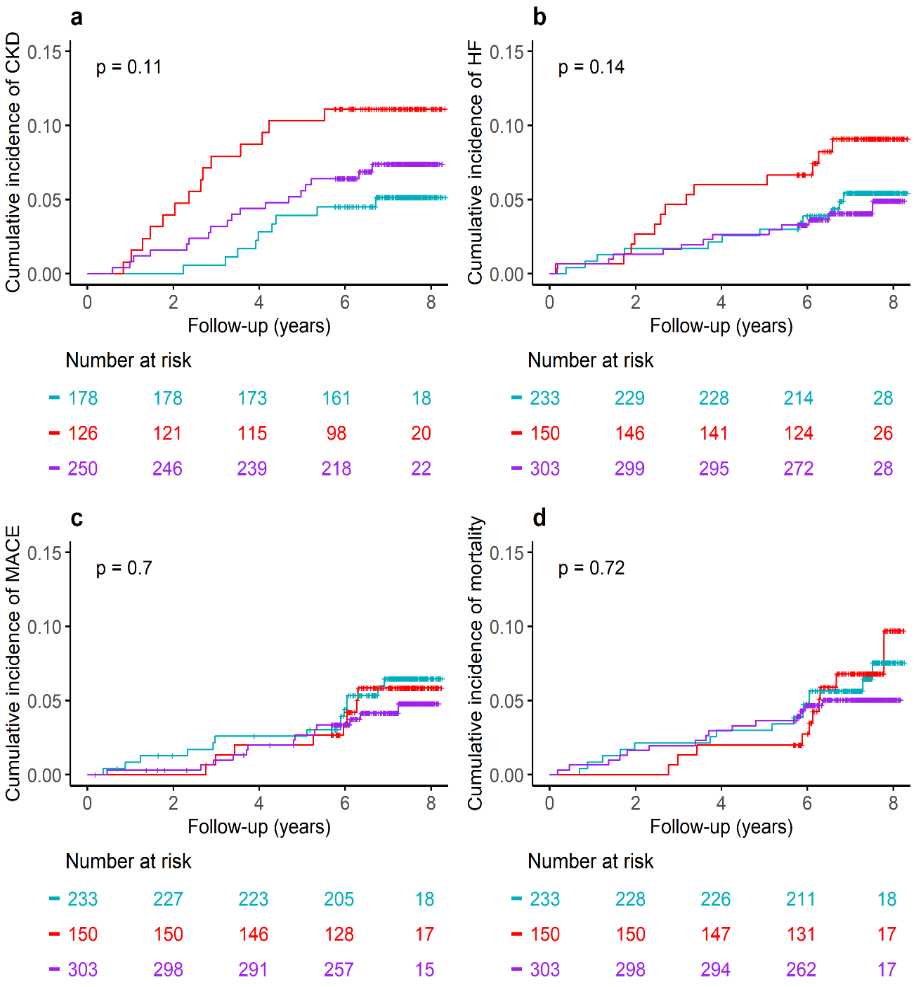

ESM Figure 6: Cumulative risk for cardio-renal events in clusters classified by diabetes onset age, BMI, HbA1c and Triacylglycerol/HDL cholesterol ratio
